# Case report: Managing pemphigus foliaceus using apremilast without systemic glucocorticosteroids or immunosuppressive agents

**DOI:** 10.3389/fimmu.2024.1408116

**Published:** 2024-07-30

**Authors:** Quanhong Zhang, Lang Yu, Li Wan, Liuqing Chen, Jinbo Chen

**Affiliations:** ^1^ Department of Dermatology, Wuhan No.1 Hospital, Wuhan, China; ^2^ Department of Dermatology, Traditional Chinese and Western Medicine Hospital of Wuhan, Tongji Medical College, Huazhong University of Science and Technology, Wuhan, China; ^3^ Jianghan University School of Medicine, Wuhan, China; ^4^ Dermatology Hospital of Southern Medical University, Guangzhou, China; ^5^ Hubei Province & Key Laboratory of Skin Infection And Immunity, Wuhan, China

**Keywords:** pemphigus foliaceus, apremilast, regulatory T cells, autoimmunity, bullous disease

## Abstract

Pemphigus foliaceus (PF) is a superficial form of pemphigus. Treatment options for PF resemble pemphigus vulgaris, including glucocorticosteroids, immunosuppressive agents and rituximab et al. These treatment approaches can effectively improve the condition but may also be accompanied by high risks of side effects. Therefore, it is crucial to find a safe and effective treatment options for patients with PF. It will not only benefit/be necessary for patients who refuse glucocorticosteroids or immunosuppressive agents treatments, but also for patients who cannot be treated with glucocorticosteroids or immunosuppressive agents. Herein, we reported a case of PF that was treated with apremilast without systemic glucocorticosteroids or immunosuppressive agents. A 54-year-old woman presented with itchy erythema and erosions on the trunk for more than 1 month. The patient applied mometasonefuroate cream without improvement for a duration of two weeks. The past history of diabetes mellitus and atrophic gastritis was reported. Physical examination revealed scattered erythematous macules and erosions on the trunk. No mucosal involvement was observed. The condition was assessed by the pemphigus disease area index and numerical rating scale, with baseline scores of 7 and 8, respectively. Histopathological examination showed acantholysis and intraepithelial blister. Direct immunofluorescence revealed the presence of IgG and Complement 3 deposition between the acanthocytes with the reticular distribution. Based on enzyme-linked immunosorbent assay results, the levels of Dsg1 and Dsg3 antibodies were 28.18 and 0.26 kU/L respectively. The diagnosis of PF was made. This patient was successfully treated with apremilast without systemic glucocorticosteroids or immunosuppressive agents. The patient has continued with apremilast 30mg once daily for maintenance and no adverse events related to apremilast such as gastrointestinal side effects were observed during the 9-month follow-up period. In conclusion, apremilast therapy without systemic glucocorticosteroids nor immunosuppressive agents might provide an effective alternative to management of mild PF without obvious side effect.

## Background

Pemphigus foliaceus (PF) is a superficial variant of pemphigus and shares similar treatment modalities with pemphigus vulgaris (PV), such as systemic glucocorticosteroids, immunosuppressive agents and rituximab and so on (1). While these treatments have demonstrated efficacy in improving the condition, they are also associated with a notable risk of side effects (1). Therefore, the exploration of safe and efficacious therapeutic alternatives for PF patients is imperative. This is particularly relevant for individuals who are averse to glucocorticosteroids or immunosuppressive agents, as well as those who are contraindicated for such treatments. Apremilast, a small molecule PDE-4 inhibitor, has demonstrated efficacy in modulating inflammatory responses and has been utilized in the treatment of psoriasis and behcet’s disease (2, 3). Furthermore, one case each of successful treatment of PV and IgA pemphigus with apremilast have been reported. In this study, we present a case of PF successfully treated with apremilast without the use of systemic glucocorticosteroids or immunosuppressive agents.

## Case presentation

**Figure 1:**
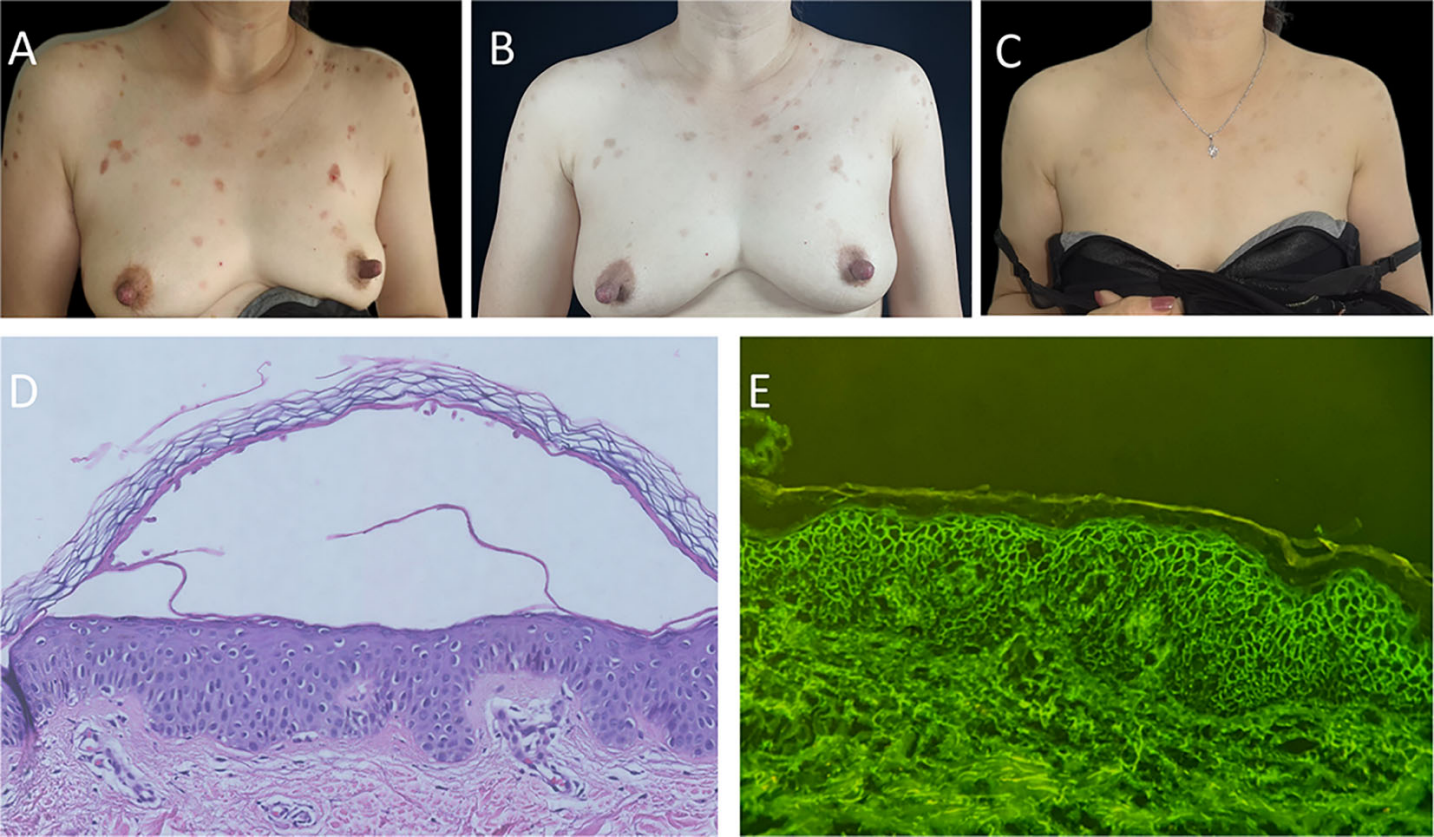
Clinical changes of the patient’s trunk, histopathology, and immunofluorescence findings. **(A)** Anterior chest of patient with scattered erythema and erosion before apremilast therapy. **(B)** Previous erythema on the anterior chest had darkened or was fading after 1-month apremilast therapy. **(C)** Darkened erythema on the anterior chest of patient after 9-month apremilast therapy. **(D)** Histopathological findings revealed acantholysis and intraepithelial blister (hematoxylin and eosin staining, ×40). **(E)** Direct immunofluorescence revealed the presence of IgG deposition between the acanthocytes with the reticular distribution (original magnification, ×200).

A 54-year-old woman presented with pruritic erythema and erosions on the trunk persisting for more than 1 month. The lesions had not improved with a two-week course of mometasone furoate cream. Her past medical history included diabetes mellitus and atrophic gastritis. Physical examination revealed scattered erythematous macules and erosions on the trunk (**Figure 1A**), with no signs of oral mucosal involvement. The severity of the condition was evaluated using the pemphigus disease area index (PDAI) and numerical rating scale (NRS), yielding baseline scores of 7 and 8, respectively. Histopathological examination revealed acantholysis and intraepithelial blister (**Figure 1D**), while direct immunofluorescence demonstrated the presence of IgG (**Figure 1E**) and Complement 3 (C3) deposition between the acanthocytes in a reticular pattern. Based on the results of enzyme-linked immunosorbent assay (ELISAs), the levels of anti-Dsg1 and anti-Dsg3 antibodies (MBL, Nagoya, Japan) were 28.18 and 0.26 kU/L (normal value <9 kU/L) respectively. The diagnosis of PF was made. Due to the patient’s medical history of diabetes mellitus and atrophic gastritis, systemic glucocorticosteroids or immunosuppressive agents were not preferred. In light of previous studies on the efficacy of apremilast, the patient opted for the treatment with apremilast 30 mg twice daily in combination with halometasone cream after obtaining informed consent. After completing a 1-month course of apremilast therapy, the patient's condition exhibited stability with the absence of new lesions and a darkening of previous erythema (**Figure 1B**). Additionally, there was a decrease in the PDAI score descended from 7 to 1, and the NRS score from 8 to 0. The patient has continued therapy with apremilast 30 mg twice daily and the levels of anti-Dsg1 antibodies decreased from 28.18 to 1.48 kU/L following the 3-month follow-up. Following this, the patient was prescribed apremilast 30mg once daily for maintenance until the 9-month follow-up period, during which time no relapses were reported and darkened erythema was observed on the anterior chest (**Figure 1C**). Furthermore, no adverse events related to apremilast, such as gastrointestinal side effects, were noted. Notably, there was an increase in the number of regulatory T cells (Treg) and induced regulatory T cells (iTreg) post-treatment, reaching 8.15 % and 9.59 %, respectively (normal value 0.1-2.05 % and 0.6-5.09 %).

## Methods

### Detection of anti-desmoglein autoantibodies

The presence of immunoglobulin G (IgG) autoantibodies directed against Dsg1 or Dsg3 in patient’s sera was evaluated by anti-Dsg1- and anti-Dsg3-ELISA according to the manufacturer’s protocol (MBL, Nagoya, Japan). Antibody positivity was defined as ELISA levels of >20KU/L for both anti-Dsg1 and anti-Dsg3.

### Histology and immunofluorescence

Skin biopsy samples were collected for the purpose of conducting direct histologic analyses and immunofluorescence. Cryocut sections from the patient’s skin or mucous membranes were separately incubated with optimally diluted FITC-labeled monospecific immunoglobulins (IgG, IgA, and IgM) and C3 for staining.

### Flow cytometric analysis

The serum samples of the patient were subjected to immunological monitoring to assess the impact on peripheral circulating T cells through flow cytometric analysis to evaluate T cells function. Tests were conducted to evaluate T cell function, including cytotoxic T lymphocyte (CTL), helper T cell (Th) and Treg cells. Regulatory T cells (Tregs) identified by the markers CD4^+^, CD25^+^ and CD127^low^. iTreg identified by the markers CD4^+^, CD25^+^, CD127^low^ and CD45RO^+^. B cells were identified using CD19 markers in Fluorescence Activated Cell Sorting (FACS). Instrument operation and data collection followed the guidelines provided by the manufacturer (Navios, Beckman Coulter,USA). The experimental data was analyzed using appropriate software (Kaluza, Beckman Coulter, USA).

## Discussion

Meier et al. reported the first case of therapy-resistant PV successfully treated with a regimen of prednisolone 20 mg, mycophenolate mofetil 2 g, and apremilast 60 mg daily. The patient’s original oral cavity erosion exhibited complete healing only after a 7-month course of treatment (4). In this particular case, the efficacy of apremilast appeared to be modest and gradual. Conversely, our study demonstrated the effectiveness of apremilast as a standalone treatment without the use of systemic glucocorticosteroids or immunosuppressive agents after just one month. Nevertheless, the precise mechanism of action of apremilast in the treatment of this disease remains unclear. Pemphigus is recognized as a Treg-associated autoimmune disease (5). Treg cells have the capacity to exert suppressive effects under specific conditions. It has been established that cAMP plays a crucial role in Treg cell-mediated suppression (6). Apremilast, by preventing the degradation of cAMP to 5’-AMP and increasing cAMP levels, can effectively modulate a wide range of inflammatory mediators, thereby offering a promising therapeutic approach for chronic inflammatory skin diseases. Apremilast has the potential to inhibit the production of interleukin-8 (IL-8), resulting in reduced neutrophil infiltration and preservation of epidermal integrity (7). Furthermore, according to Sigmund et al., apremilast has been shown to counteract pemphigus autoantibody-induced loss of keratinocyte adhesion through the elevation of cAMP levels, thereby potentially preventing blister formation and promoting the stabilization of keratinocyte adhesion (8). Consequently, it is postulated that the observed clinical improvements in our case following apremilast treatment may be linked to these mechanisms. In a recent study by Yuxi et al., a case of refractory atypical IgA pemphigus demonstrated a favorable response to apremilast, suggesting the potential of apremilast as an innovative therapeutic option for resistant cases of IgA pemphigus (9). In conclusion, apremilast therapy, in the absence of systemic glucocorticosteroids or immunosuppressive agents, may offer a viable alternative for managing mild PF with minimal side effects. This treatment option could be particularly beneficial for patients with mild PF who are unwilling to or unable to undergo treatment with systemic glucocorticosteroids or immunosuppressive agents. Nevertheless, further investigation with extended follow-up periods and larger sample sizes is necessary to validate our results and monitor for potential relapses.

## Data availability statement

The original contributions presented in the study are included in the article/supplementary material. Further inquiries can be directed to the corresponding authors.

## Ethics statement

The studies involving humans were approved by Ethics Committee of Wuhan No. 1 Hospital. The studies were conducted in accordance with the local legislation and institutional requirements. Written informed consent for participation was not required from the participants or the participants’ legal guardians/next of kin in accordance with the national legislation and institutional requirements. Written informed consent was obtained from the individual(s) for the publication of any potentially identifiable images or data included in this article.

## Author contributions

QZ: Writing – original draft. LY: Writing – original draft. LW: Writing – original draft. LC: Writing – review & editing. JC: Writing – review & editing.

## References

[1] Borradori L Van Beek N Feliciani C Tedbirt B Antiga E Bergman R. Updated S2 K guidelines for the management of bullous pemphigoid initiated by the European Academy of Dermatology and Venereology (EADV). J Eur Acad Dermatol Venereol. (2022) 36:1689–704. doi: 10.1111/jdv.18220 35766904

[2] Armstrong AW Gooderham M Warren RB Papp KA Strober B Thaçi D. Deucravacitinib versus placebo and apremilast in moderate to severe plaque psoriasis: Efficacy and safety results from the 52-week, randomized, double-blinded, placebo-controlled phase 3 POETYK PSO-1 trial. J Am Acad Dermatol. (2023) 88:29–39. doi: 10.1016/j.jaad.2022.07.002 35820547

[3] Hatemi G Mahr A Ishigatsubo Y Song YW Takeno M Kim D. Trial of apremilast for oral ulcers in behçet’s syndrome. N Engl J Med. (2019) 381:1918–28. doi: 10.1056/NEJMoa1816594 31722152

[4] Meier K Holstein J Solimani F Waschke J Ghoreschi K. Case report: apremilast for therapy-resistant pemphigus vulgaris. Front Immunol. (2020) 11:588315. doi: 10.3389/fimmu.2020.588315 33193415 PMC7653172

[5] Xu M Liu Q Li S Zhang W Huang X Han K. Increased expression of miR-338-3p impairs Treg-mediated immunosuppression in pemphigus vulgaris by targeting RUNX1. Exp Dermatol. (2020) 29:623–9. doi: 10.1111/exd.14111 32386260

[6] Klein M Bopp T. Cyclic AMP represents a crucial component of treg cell-mediated immune regulation. Front Immunol. (2016) 7:315. doi: 10.3389/fimmu.2016.00315 27621729 PMC5002888

[7] Schafer P. Apremilast mechanism of action and application to psoriasis and psoriatic arthritis. Biochem Pharmacol. (2012) 83:1583–90. doi: 10.1016/j.bcp.2012.01.001 22257911

[8] Sigmund AM Winkler M Engelmayer S Kugelmann D Egu DT Steinert LS. Apremilast prevents blistering in human epidermis and stabilizes keratinocyte adhesion in pemphigus. Nat Commun. (2023) 14:116. doi: 10.1038/s41467-022-35741-0 36624106 PMC9829900

[9] Zhou Y Xiao Y Wang Y Li W. Refractory atypical IgA pemphigus successfully treated with apremilast. J Dermatol. (2024) 51:e86–e7. doi: 10.1111/1346-8138.17007 37864455

